# Surgical Anatomy of the Mesopancreas in Obese Patients: A Retrospective Study

**DOI:** 10.7759/cureus.37806

**Published:** 2023-04-18

**Authors:** Amer Q Aldouri, Zergham Zia, Munhal Agouba, Salman Almaliki, Badr Bannan, Nada Majeed, Maha D Alfandi, Majed Ashour, Gareth Morris-Stiff, Zain Alshareef

**Affiliations:** 1 Surgery, King Faisal Specialist Hospital and Research Centre, Al-Madinah Al-Munawwarah, SAU; 2 Radiology, King Faisal Specialist Hospital and Research Centre, Jeddah, SAU; 3 Surgery, King Faisal Specialist Hospital and Research Centre, Jeddah, SAU; 4 Surgery, Cleveland Clinic Lerner College of Medicine of Case Western Reserve University, Cleveland, USA

**Keywords:** superior mesenteric artery, celiac axis, topographical anatomy, pancreatic cancer resection, mesopancreas

## Abstract

Introduction

The mesopancreas is described as a triangle formed by the superior mesenteric vein, celiac axis (CA), and superior mesenteric artery (SMA). It is the most likely site of residual cancer and local recurrence after surgical resection, making it the key site of the current radical resection of pancreatic head cancer. The surgical anatomy of the mesopancreas triangle has not been studied in detail. Furthermore, to the best of our knowledge, no information is available on the impact of obesity on the anatomy of the mesopancreas triangle.

Methods

Between January 2016 and August 2016, 200 consecutive triple-phase computed tomography scans of the abdomen were performed and included in this retrospective study aiming to define the anatomical relation of the left renal vein (LRV) to the root of the SMA and focusing on the relevance of the LRV as a landmark to guidance for the dissection of the mesopancreas. Furthermore, by studying six surgically relevant anatomical parameters namely the thickness of the areolar tissue separating the LRV from the root of the SMA, IVC from the root of the SMA, the left adrenal vein (LAV) from the root of the SMA, splenic vein from the aorta, and CA from the SMA at two levels, we investigated the impact of obesity on the mesopancreas anatomy.

Results

The mean distance from the upper border of the LRV to the root of the SMA (LRV-SMA distance) was 2.3 ± 5.4 mm. There was no correlation between this distance and patient’s age (*r* = -0.02), height (*r* = -0.07), BMI (*r* = -0.01), visceral fat area (*r* = -0.04), or abdominal circumference (*r* = -0.02). There was no correlation between the distance from the IVC to the root of the SMA, and patient’s age (*r* = 0.01), height (*r* = 0.11), BMI (*r* = 0.15), or abdominal circumference (*r* = 0.00). However, there was a negligible correlation between the IVC-SMA distance and patient’s visceral fat area (*r* = 0.15, *p *= 0.036).

Conclusion

In the current study, the LRV was reliably identified in more than 99% of the studied patients, and in 96% of patients, the LRV crosses anterior to the aorta at the level of the second lumbar vertebra, making it easily accessible following mobilization of the duodenum and the head of the pancreas. The relationship between the LRV and SMA remains unchanged following Kocherization. Most importantly, we demonstrated that the LRV-SMA distance does not correlate with patient’s age, height, BMI, visceral fat area, or abdominal circumference. This makes the LRV a reliable landmark in both obese and non-obese patients.

## Introduction

The mesopancreas, as described by Adham and Singhirunnusorn, is a triangle with its base lying on the posterior surface of the superior mesenteric vein (SMV) and portal vein (PV), with the apex lying on the anterior surface of the aorta between the celiac axis (CA) and superior mesenteric artery (SMA) origin, and its lateral boundaries limited by the right semi-circumferences of the CA and SMA plexus [1]. The authors summarized the technique for total mesopancreas excision (TMpE) commencing with mobilization of the pancreatic head to allow identification of the SMA above the distal portion of the left renal vein (LRV), just above the angle created by the junction between the LRV and inferior vena cava (IVC). A right-sided semi-circumferential exposure of the SMA is then performed with dissection conducted along the anterior surface of the aorta, this maneuver exposes the origin of the CA. 

The mesopancreas is the most likely site of residual tumors and local recurrence after surgical resection of the pancreatic head tumor, making it the key site of radical resection pancreatic head cancer [2]. There is currently a lack of well-defined data confirming the landmarks of the mesopancreas [3]. The surgical anatomy of the mesopancreas triangle has not been studied in detail. Furthermore, to the best of our knowledge, no information is available on the impact of obesity on the anatomy of the mesopancreas triangle.

We conducted this study to examine the reliability of the potential landmarks described by Adham and Singhirunnusorn in both obese and non-obese patients and to delineate the anatomy of the mesopancreas triangle.

## Materials and methods

Inclusion criteria 

All kidney transplant donors who had triple-phase computed tomography (CT) scans of the abdomen as part of the donor assessment protocol between January 2016 and August 2016 were included.

Exclusion criteria

The exclusion criteria were as follows: (1) all cases with LRV anomaly (type one anomaly, obliterated preaortic LRV with persistent retro-aortic LRV at the level of second lumbar vertebra, type two anomaly, obliterated preaortic LRV with persistent retroaortic LRV at the level of 4th to 5th lumbar vertebra, type three anomaly is circum-aortic LRV, or venous collar, and type four anomaly, the LRV is joining the left iliac vein) and (2) all cases where the LRV was not identified.

Methods

Between January 2016 and August 2016, 200 consecutive triple-phase CT scans of the abdomen were performed in our institution as part of a prerenal transplant donation protocol. Written informed consent was not required to review these CT scans for this retrospective study, in accordance with guidance from our Local Research Ethics Committee. All abdominal imaging figures used in this report are from the study participants.

All patients were examined in the supine position on a 64-slice multi-detector CT machine (Siemens Somatom Sensation, Siemens AG, Forchheim, Germany). Up to 100 ml (2 mg/kg) of intravenous contrast medium (Iomeron 300; Bracco, High Wycombe, UK) was administered. Arterial and venous phase images were available in all cases and provided confident identification of the SMA, LRV, CT, left adrenal vein (LAV), and IVC. 

The CT scan images included in the study were interpreted by three experienced radiologists (one senior consultant and two senior residents) and one consultant hepato-pancreatico-biliary surgeon using a radiology workstation monitor (Agfa HealthCare, Ghent, Belgium). The radiologists were blinded to the radiology reports. 

In this study, the anatomical relation of the LRV to the root of the SMA (the proximal 10 mm of the SMA) was determined, focusing on the relevance of the LRV as a landmark to guidance for the dissection of the mesopancreas. Furthermore, this study examined the impact of obesity on the anatomy of the mesopancreas, by studying six surgically relevant anatomical parameters namely the thickness of the areolar tissue separating the LRV from the root of the SMA, IVC from the root of the SMA, LAV from the root of the SMA, splenic vein (SpV) from the aorta, and CA from the SMA at two levels (at the level of the LRV and the level of the common hepatic artery origin (CHA)). Each of these parameters was examined in relation to the patient’s age, height, BMI, abdominal visceral fat area, and abdominal circumference. 

**Figure 1:**
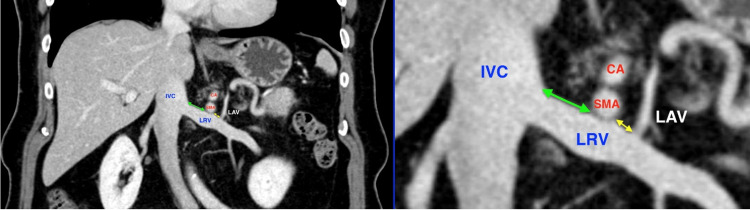
Coronal CT scan image showing the relationship between the LRV and the root of the SMA. The distance from the IVC to the root of the SMA is marked by the green arrow, and the distance from the LAV to the root of the SMA is marked by the yellow arrow. LRV: Left renal vein; SMA: superior mesenteric artery; CA: celiac axis; IVC: inferior vena cava; LAV: left adrenal vein; CT: computed tomography

Axial slices were used to identify the LRV and the origin of the SMA and cross-referenced to sagittal reconstructions. The LRV-SMA distance was measured as the nearest distance from the LRV superior wall to the SMA inferior wall. The measurements undertaken are shown in Figure 1. 

Statistical analysis was performed using Student’s t-test and Pearson’s correlation coefficient, with significance assumed at the 5% level, using IBM SPSS Statistics for Windows, Version 20 (Released 2011; IBM Corp., Armonk, New York, United States). The correlation coefficient was interpreted as negligible (0.0 to ± 0.30), low correlation (0.30 to 0.50), moderate correlation (0.50 to 0.70), strong (0.70 to 0.90), or very strong (0.90 to 1.00) [4].

## Results

The study cohort consisted of 50 females and 150 males, with a mean age of 31 years (range, 17-53 years). The mean BMI was 27.01 ± 4.89 (range, 16.6-39.7) kg/m^2^. Of the 200 patients, 70 (35%) were normal weight, 71 (35%) were overweight, and 59 (30%) were obese. 

Of the consecutive 200 CTs selected for LRV analysis, eight patients were excluded for the following reasons: in seven patients, the LRV was crossing the aorta in an unusual pattern (in four patients at level caudal to the second lumbar vertebral body and in three cases, the LRV had a retroaortic course), and in one case, the LRV was not observed, thus leaving 192 patients for LRV analysis. The SMA and CA were observed in all the patients assessed. In two patients, the SMA and CA have a common trunk that arises from the aorta. All measurements are summarized in Table 1. 

**Table 1:** Summary of the relationships between anatomical measurements and age, patient height, body mass index, visceral fat area, and abdominal circumference. r: correlation coefficient; p: statistical significance; *: statistically significant; **: positive relationship.

	Statistics	Age	Height	BMI	Visceral fat area	Abdominal circumference
LRV-SMA distance	r ( p)	-0.02 (0.75)	-0.07 (0.32)	-0.01 (0.86)	-0.04 (0.56)	-0.02 (0.69)
IVC-SMA distance	r (p)	0.01 (0.88)	0.11 (0.11)	-0.01 (0.83)	0.15 (0.03*)	0.00 (0.89)
LAV-SMA distance	r (p)	-0.05 (0.46)	0.04 (0.50)	-0.03 (0.66)	0.06 (0.35)	-0.02 (0.75)
CA-SMA distance at level of LRV	r (p)	-0.08 (0.24)	0.01 (0.88)	0.28 (<0.00*)	0.26( <0.00*)	0.29 (<0.00*)
CA-SMA distance at level of CHA	r (p)	0.16 (0.02*)	0.04 (0.57)	0.23 (<0.00*)	0.34** (<0.00*)	0.27 (<0.00*)
SpV-Aortic distance	r (p)	0.29 (<0.00*)	0.15 (0.03*)	0.20 (<0.00*)	0.48** (<0.00*)	0.32** (<0.00*)

LRV relation to SMA

The mean distance from the upper border of the LRV to the root of the SMA (LRV-SMA distance) was 2.3 ± 5.4 mm. No sex differences were observed (p = 0.15). The LRV-SMA distance was examined in relation to the patient’s age, height, BMI, abdominal visceral fat area, and abdominal circumference. There was no correlation between this distance and patient’s age (r = -0.02), height (r = -0.07), BMI (r = -0.01), visceral fat area (r = -0.04), or abdominal circumference (r = -0.02). 

IVC relation to SMA

The mean distance from the medial border of the IVC to the root of the SMA (IVC-SMA distance) was 13.41 ± 4.10 mm (range: 1.5 - 21.6 mm); this distance was significantly longer in male patients compared to female patients (13.9 vs 12.0 mm, p = 0.02). The IVC-SMA distance was examined in relation to the patient’s age, height, BMI, abdominal visceral fat area, and abdominal circumference. There was no correlation between this distance and patient’s age (r = 0.01), height (r = 0.11), BMI (r = 0.15), or abdominal circumference (r = 0.00). However, there was a negligible correlation between the IVC-SMA distance and patient’s visceral fat area (r = 0.15, p = 0.036). 

LAV relation to the SMA

The mean distance from the LAV to the root of the SMA (LAV-SMA distance) was 8.39 ± 5.39 mm (range; 0 - 52.0 mm); sex differences were not observed (p = 0.69). The LAV-SMA distance was examined in relation to the patient’s age, height, BMI, abdominal visceral fat area, and abdominal circumference. There was no correlation between this distance and patient’s age (r = -0.05), height (r = 0.04), BMI (r = -0.03), visceral fat area (r = 0.06), or abdominal circumference (r = -0.02). 

CA relation to the SMA at the level of the LRV 

The mean distance from the CA to the SMA at the level of the LRV (CA-SMA at LRV distance) was 4.71 ± 2.99 mm (range: 0.0 - 13.4 mm); sex differences were not observed (p = 0.70). The CA-SMA at LRV distance was examined in relation to the patient’s age, height, BMI, abdominal visceral fat area, and abdominal circumference. There was no correlation between this distance and patient’s age (r = 0.08) or height (r = 0.01). However, there was a negligible linear correlation with the patient’s BMI (r = 0.28, p < 0.000), visceral fat area (r = 0.26, p < 0.000), and abdominal circumference (r = 0.29, p < 0.000). 

CA relation to the SMA at the level of the CHA 

The mean distance from the CA to the SMA at the level of the CHA was 10.35 ± 6.03 mm (range: 1.7 - 28.4 mm); sex differences were not observed (p = 0.28). The CA-SMA at CHA distance was examined in relation to the patient’s age, height, BMI, abdominal visceral fat area, and abdominal circumference. There was no correlation between this distance and patient’s height (r = 0.04). However, there was correlation between this distance and patient’s age (r = 0.16, p = 0.02), BMI (r = 0.23, p = 0.001), visceral fat area (r = 0.34, p < 0.000), and abdominal circumference (r = 0.27, p < 0.000). 

SpV relation to the aorta 

The mean distance from the SpV to the anterior aortic wall was 18.93 ± 5.61 mm (range: 8.037.5 mm). The mean SpV-aortic distance was significantly longer in male than female patients (19.9 versus 15.9, p < 0.000). This distance was examined in relation to patient’s age, height, BMI, abdominal visceral fat area, and the abdominal circumference. There was correlation between this distance and patient’s age (r = 0.29, p < 0.00), height (r = 0.15, p = 0.03), BMI (r = 0.20, p < 0.00), visceral fat area (r = 0.48, p < 0.00), and abdominal circumference (r = 0.32, p < 0.00). 

Aberrant right hepatic artery 

In 23 patients (11%), an aberrant right hepatic artery (aRHA) was identified. In 21 patients (10.5%), the aRHA arose from the SMA. The mean distance from the root of the SMA to the origin of the aRHA was 19.52 ± 3.87 mm (range: 13-32 mm). 

Inferior right phrenic artery (IRPA)

The IRPA runs on the ventral surface of the right diaphragmatic crus in close relation to the CA and SMA origin from the aorta. IRPA could be detected in 193 of 200 patients. We determined three patterns of the IRPA in relation to SMA and CA origins. In the majority of patients (n = 97, 50%), it arose at the level of CA and ran laterally cranial to the CA. In 67 patients (35%), the IRPA ran to the right of the CA or SMA origin, and in a further 29 patients (15%), the IRPA ran caudally to the SMA origin. 

Dorsal pancreatic artery (DPA)

The DPA could be detected in 182 of 200 patients (91%). It ran between the CA and SMA, posterior to the splenic vein close to the spleno-portal confluence. No further significant vessels were identified between the CA and SMA or to the right of them. 

## Discussion

This is a cross-sectional study to report the detailed anatomy of the mesopancreas in relation to obesity and abdominal visceral fat area. Adham and Singhirunnusorn summarized the technique for TMpE [1]. However, their innovative technique did not address the challenges encountered during performing TMpE for obese patients or the feasibility of this technique in the presence of aberrant arterial anatomy. 

CT images provide surgeons with accurate details of the anatomic settings they may face during surgical procedures. Anatomical landmarks help to guide the operating surgeon to perform complex procedures, such landmarks should fulfill the following criteria: occurring with a high incidence, being easy to identify, being easily accessible, and being resistant to manipulation during surgery [5]. 

In the current study, the LRV was reliably identified in more than 99% (199 of 200) of the studied patients, and in 96% (192 of 200) of patients, the LRV crosses anterior to the aorta at the level of the second lumbar vertebra, making it easily accessible following mobilization of the duodenum and the head of the pancreas. The relationship between the LRV and SMA remains unchanged following Kocherization. Most importantly, we demonstrated that the LRV-SMA distance does not correlate with patient’s age, height, BMI, visceral fat area, or abdominal circumference. This makes the LRV a reliable landmark in both obese and non-obese patients. 

Referring to the angle of the LRV’s confluence with the IVC as a landmark, surgeons can estimate with reliable accuracy the position of the root of the SMA. Preoperative measurement of IVC-SMA distance helps to precisely identify the nearest point of LRV to the root of the SMA. Furthermore, preoperative measurement of the LRV-SMA distance identifies the thickness of the areolar tissue separating the LRV from the root of the SMA. Both will guide the surgeon to a reliable window for accessing the SMA. In this study, we showed that the mean distance from the medial wall of IVC to the root of the SMA did not correlate with any of the variables assessed, and so this fact supports the reliability of the angle between the LRV confluence and the IVC as a landmark in both obese and non-obese patients. 

Following the identification of the root of the SMA, the surgeon can use the SMA as a reference point to access the CA. The distance from the SMA to the CA at the level of the LRV serves as a guide to identifying the CA. We identified a negligible correlation between this distance and patient’s BMI (r = 0.28), visceral fat area (r = 0.26), and abdominal circumference (r = 0.29). 

The distance from the SMA to the CA at the level of the CHA showed a low correlation with patient’s visceral fat area (r = 0.34). However, this is unlikely to add a degree of difficulty to the dissection as both the SMA and CA would be already identified at the level of the LRV, and they will be serving as reference points to guide dissection toward the CHA and SpV. 

**Figure 2:**
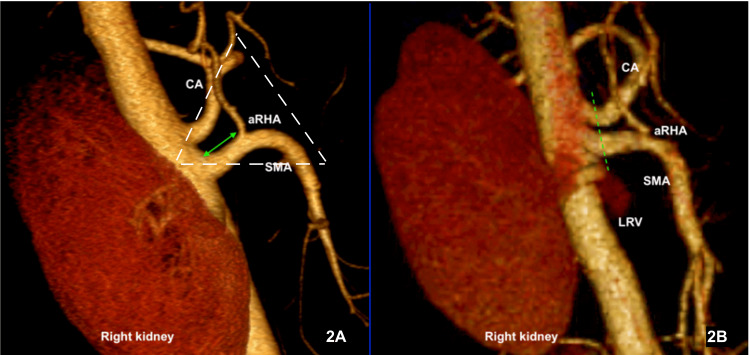
A 3D reconstruction of a CT scan image showing the relationship between the LRV and the root of the aRHA. (A) The distance from the aorta to the root of the aRHA is marked by the green arrow; the white dotted triangle represents the mesopancreas area. (B) The dissection plane at the level of the LAV denoted by the dotted green line. LRV: Left renal vein; SMA: superior mesenteric artery; CA: celiac axis; aRHA: aberrant right hepatic artery; 3D: three-dimensional; CT: computed tomography

**Figure 3:**
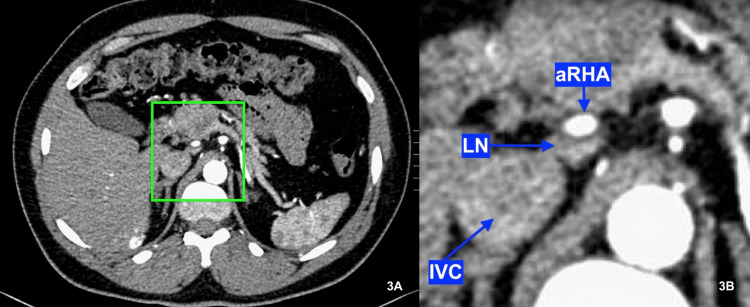
CT scan image showing the relationship between the aRHA and the related lymph nodes. (A) Axial CT image showing the aRHA arising from the SMA. (B) Magnified image of axial CT showing the main trunk of the aRHA covered posteriorly by the LN. IVC: Inferior vena cava; aRHA: aberrant right hepatic artery; LN: lymph node; CT: computed tomography

Within the boundaries of the mesopancreas triangle, the surgeon may encounter two significant arteries, an aRHA and DPA. In the current study, a little over 10% of patients had an aRHA raise from the SMA. Looking at the challenge imposed by the presence of aRHA during TMpE, namely that aRHA may be encountered during initial dissection of the SMA at the level of LRV, our study showed that the mean distance from the origin of SMA to the origin of aRHA (denoted by the green arrow in Figure 2A) was 19.52 ± 3.87 mm, suggesting that dissecting the root of SMA at the level of LRV (denoted by the green line in Figure 2B) is safe as this plane is posterior to the root and the course of aRHA. Furthermore, our study observed that the aRHA was related posteriorly to lymph nodes (Figure 3), this finding being compatible with the findings of Nakagawara and colleague’s manuscript [6]. These lymph nodes separate the aRHA from the dissection plane; however, they should be excised later as part of the standard lymphadenectomy during pancreatoduodenectomy. 

**Figure 4:**
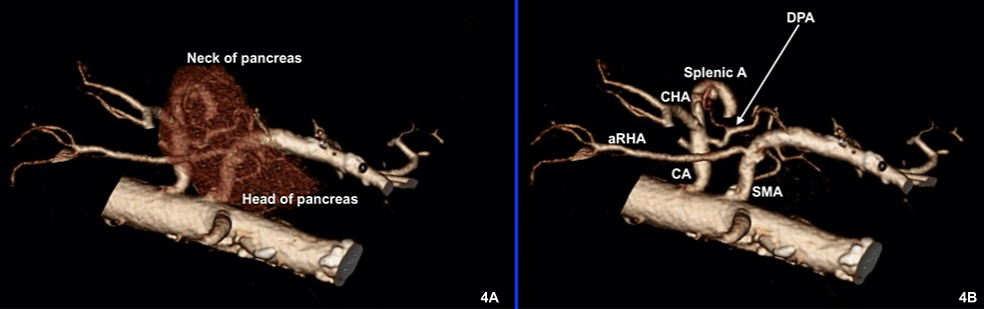
3D reconstruction of a CT scan image showing the course of both the DPA and the aRHA at the base of the mesopancreatic triangle. (A) Showing the relation of the DPA to the head of pancreas. (B) Demonstrating the relation of the DPA to the aRHA. SMA: Superior mesenteric artery; CA: celiac axis; aRHA: aberrant right hepatic artery; DPA: dorsal pancreatic artery; CHA: common hepatic artery; Splenic A: splenic artery; 3D: three-dimensional; CT: computed tomography

In this study, the DPA was observed in 91% of cases and provided a constant branch to the head of the pancreas as described previously [7]. This branch runs posterior to the SMV or PV to reach the retroportal portion of the pancreatic head, running across the base of the mesopancreatic triangle, (Figure 4). The DPA’s branch is usually encountered during TMpE and should be identified and ligated. 

Studies from Japan have suggested improved patient's survival following a TMpE for patients with ductal adenocarcinoma of the pancreas [8,9]. Furthermore, a meta-analysis study showed that the artery first approach was associated with better preoperative outcomes and less recurrence rate compared to standard pancreaticoduodenectomy [10]. The feasibility of the supracolic artery-first approach in a patient with central obesity has also been previously demonstrated [11].

**Figure 5:**
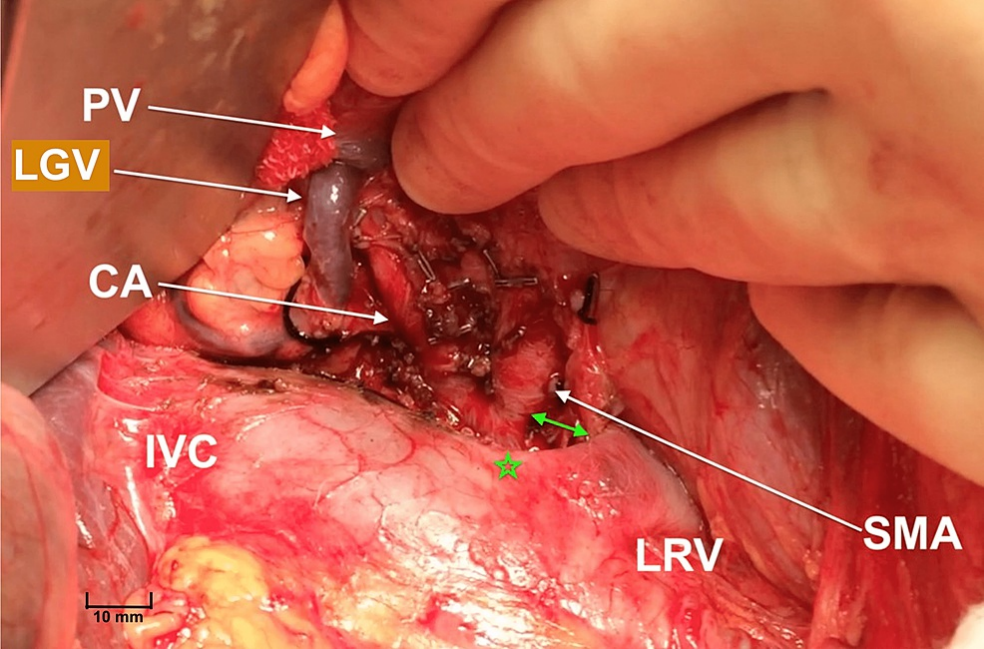
Intraoperative images showing the second stage of TMpE. In the second stage, the head of the pancreas is mobilized and the neurovascular and lymphatic areolar tissue to the right of SMA and CA is excised. The angle of the LRV confluence with the IVC (green star) is used as a landmark to identify the nearest point of the LRV to the root of the SMA (green arrow). LRV: Left renal vein; SMA: superior mesenteric artery; CA: celiac axis; IVC: inferior vena cava; PV: portal vein; LGV: left gastric vein; TMpE: total mesopancreas excision

Our approach consists of two-stage resection of the mesopancreas, starting with a supracolic artery first approach to control the arterial inflow and venous return prior to mobilization of the head of the pancreas [12]. The second stage is to mobilize the head of the pancreas and dissect the mesopancreatic triangle (Figure 5). Although Adham and Singhirunnusorn advocated en bloc resection of the mesopancreas, a staged resection approach has been shown to lead to a comparable survival to that of en bloc resection [13].

This study has many limitations including the number of examined cases, which is relatively small. Also, the study does not include patients with a BMI of more than 40 kg/m^2^. Furthermore, all patients included in this study were healthy individuals, undergoing abdominal imaging prior to kidney donation. 

That said, this the first report to examine the relationship between visceral abdominal fat area and anatomical relations among the abdominal main vessels and has reported the detailed anatomy of the mesopancreas triangle. This study has confirmed that the CA-SMA distance at the level of the CHA and the SpV-aortic distance both have a low correlation with visceral obesity. The fact that other measurements do not vary with BMI means that they are constant and reliable markers for the position of the mesopancreas even in obese patients. 

## Conclusions

In the current study, the LRV was reliably identified in more than 99% of the studied patients, and in 96% of patients, the LRV crosses anterior to the aorta at the level of the second lumbar vertebra, making it easily accessible following mobilization of the duodenum and the head of the pancreas. The relationship between the LRV and SMA remains unchanged following Kocherization. Most importantly, we demonstrated that the LRV-SMA distance does not correlate with patient’s age, height, BMI, visceral fat area, or abdominal circumference. This makes the LRV a reliable landmark to approach the SMA in both obese and non-obese patients.
